# Gastrointestinal perforation after bevacizumab: a multi-site, single-institution study with a focus on survival

**DOI:** 10.1186/s12957-023-03058-x

**Published:** 2023-06-08

**Authors:** Michael H. Storandt, Nguyen H. Tran, Christopher J. Ehret, Mina Hanna, Jacob Jochum, Michael R. Moynagh, Aminah Jatoi

**Affiliations:** 1 Department of Medicine, 200 First Street SW, Rochester, MN 55905 USA; 2 Department of Oncology, 200 First Street SW, Rochester, MN 55905 USA; 3 Department of Oncology, Mayo Clinic, 404 W. Fountain Street, Albert Lea, MN 56007 USA; 4 Department of Radiology, 200 First Street SW, Rochester, MN 55905 USA

**Keywords:** Bevacizumab, Perforation, Survival

## Abstract

**Background:**

Bevacizumab-induced gastrointestinal perforation is a rare but potentially devastating adverse event that has generated limited data on overall survival. Yet, such survival data are critical in guiding management.

**Methods:**

This multi-site, single-institution retrospective study focused on all cancer patients who had received bevacizumab and who had suffered a well-documented gastrointestinal perforation from January 1, 2004 through January 20, 2022.The main goal was to report survival outcomes; Kaplan Meier curves and Cox survival models were used for this purpose.

**Results:**

Eighty-nine patients are included in this report with a median age of 62 years (range 26–85). Colorectal cancer was the most common malignancy (*n* = 42). Thirty-nine patients underwent surgery for the perforation. Seventy-eight were deceased at the time of reporting with an overall median survival of all patients of 2.7 months (range 0–45 months), and 32 (36%) died within 30 days of perforation. In univariable survival analyses, no statistically significant associations were observed for age, gender, corticosteroid use, and time since last bevacizumab dose. However, surgically treated patients manifested a better survival (hazard ratio (HR) 0.49 (95% CI 0.31–0.78); *p* = 0.003). In multivariable analyses, surgery continued to be associated with improved survival (HR 0.47 (95% CI 0.29–0.74); *p* = 0.002), and corticosteroid use was associated with worse survival (HR 1.75 (95% CI 1.02–2.99); *p* = 0.04).

**Conclusion:**

Although gastrointestinal perforation after bevacizumab should be managed on a case-by-case basis, these descriptive survival data can help inform patients, their families, and healthcare providers as challenging management decisions arise.

## Introduction

Bevacizumab is a vascular endothelial growth factor (VEGF) recombinant monoclonal antibody inhibitor that was approved in 2004 as an antineoplastic agent by the Food and Drug Administration (FDA) [1]. Since this approval, the indications for this agent have expanded to include not only the treatment of colorectal cancer but also the treatment of non-small cell lung cancer, renal cell carcinoma, ovarian/fallopian/primary peritoneal cancer, hepatocellular cancer, recurrent glioblastoma, and cervical cancer [2–7].

Bevacizumab-induced gastrointestinal perforation is a rare but potentially devastating adverse event that occurs in approximately 1–3% of patients [7–11]. The details of the pathophysiology of bevacizumab-induced gastrointestinal tract perforation remain unknown [12]. However, because VEGF is implicated in wound healing and because bevacizumab may be implicated in tumor-induced immunosuppression, it seems plausible that its inhibition will lead to challenges with wound healing and regeneration of even healthy tissue [12, 13]. Further, the increasingly widespread use of bevacizumab and similar agents that inhibit VEGF place more patients at risk for this complication [2–7]. Although older age with concurrent morbidity such atherosclerosis, hypertension, and diabetes might predispose to ischemia within the gastrointestinal tract, bevacizumab-induced perforation appears to be noteworthy because of bevacizumab’s specific effects on vasculature and healing and because of the consequential established risk of perforation of the gastrointestinal tract [14]. Other pathophysiologic factors have been speculated to predispose bevacizumab-treated patients to perforation [15]. These include VEGF inhibition that (1) inhibits or induces clotting factors, leads to thrombosis of small splanchnic or mesenteric vessels, and thereby results in bowel ischemia and perforation; (2) modulates nitrous oxide, prostacyclin, and platelet function, all of which could affect microcirculation within bowel and thereby predispose to perforation; (3) carries antineoplastic effects with the death of cancer cells and disruption of bowel integrity; and (4) exacerbates small extant ulcers within the gastrointestinal tract with resulting perforation [15].

Similarly, risk factors for bevacizumab-induced gastrointestinal perforation include colon surgery within 2 months of drug administration, peptic ulcer disease, non-steroidal anti-inflammatory use, acute diverticulitis, intra-abdominal abscess, intestinal obstruction, cancer at the site of perforation, peritoneal carcinomatosis, and prior abdominopelvic radiation therapy [16]. In fact, the FDA advises delay of surgery beyond 28 days of drug administration because of this risk [7]. To our knowledge, however, few studies have focused on overall survival after perforation, and few have sought to explore clinical factors associated with overall survival [17–19].

This study sought to report survival outcomes of cancer patients who had suffered a gastrointestinal perforation after receipt of intravenous bevacizumab. It also sought to explore risk factors that might predict higher or lower risk of mortality.

## Methods

### Overview

The Mayo Clinic Institutional Review Board (IRB) reviewed and approved the study protocol (#22–000229). Informed consent of patients was waived because of the high likelihood of deceased patients.

A search of the Mayo Clinic electronic medical record generated a list of adult patients who had received bevacizumab between January 1, 2004 through January 20, 2022 and who had suffered a subsequent gastrointestinal perforation. Gastrointestinal perforation was demonstrated based on computerized tomographic (CT) scans, other radiographic evidence, or a combination of such evidence (CT scan evidence and surgical findings). The final list was comprised of patients from across the Mayo Clinic, including patients from Minnesota, Arizona, Florida, Wisconsin, and Iowa.

### Medical record review

One investigator (MHS) reviewed the medical records of all cancer patients who met the above criteria, and other investigators performed sporadic spot checks to confirm data accuracy. Each medical record was reviewed in detail for confirmation of bevacizumab administration for a cancer indication and for the occurrence of a gastrointestinal perforation, as documented in the medical record. Thereafter, further information on patients’ date of birth, sex, cancer type, prior chemotherapy exposure, date of bevacizumab administration, date of perforation, concomitant corticosteroid use, specifics related to the perforation including management, and date of death or last follow up were abstracted from the medical record.

### Analyses

The data were locked for analysis on March 22, 2022. Data are reported descriptively with averages, percentages, hazard ratios, and 95% confidence intervals (CI’s). Survival was defined as the time from date of documented perforation until death or last follow up. Survival data were censored on date of last patient contact. The distribution of this time-to-event end point was estimated by Kaplan–Meier curves. Cox proportional hazards models were used to assess the association between age at perforation, gender, corticosteroid use, time from last bevacizumab dose, and whether surgery was undertaken with respect to overall survival. A *p* value of less than 0.05 was considered statistically significant. JMP 16.1.0 was used for all analyses (NC, USA).

## Results

### Patient demographics

A total of 89 patients were identified and are the subject of this report. The median age at perforation was 62 years (range 26–85). Sixty-one patients (69%) were women. The most common cancer type was colorectal cancer, as diagnosed in 42 patients (47%). Sixty-six patients (74%) were receiving chemotherapy beyond first-line therapy. Baseline demographics and other clinical characteristics appear in Table 1.

**Table 1:** Demographics (*n* = 89)

Variable	Number (%)*
Median age at perforation in years (range)	62 (26, 85)
Gender	
Male	28 (31)
Female	61 (69)
Cancer	
Colorectal	42 (47)
Ovarian	22 (25)
Brain	8 (9)
Lung	4 (4)
Breast	2 (2)
Cervical	2 (2)
Other	9 (10)
Systemic therapy at perforation	
Only first-line	23 (26)
Beyond first-line	66 (74)
On corticosteroids at perforation	
Yes	23 (26)
No or unknown	66 (74)

^*^Numbers refer to number of patients and parentheses refer to percentages unless otherwise specified

### Information pertinent to the perforation

Only 21 patients (24%) manifested a perforation at their primary tumor site. In the remaining patients, the site of perforation was either elsewhere or unable to be definitively discerned. The most common site of perforation was the colorectal area, as seen in 46 patients (52%) (Table 2). Thirty-nine patients (44%) underwent surgery: ostomy formation in 25; over-sewing the perforation site in 6; exploration, lavage, and drain placement in 5; resection with anastomosis in 1; omentoplasty in 1; and laparotomy with no other intervention in 1.

**Table 2:** Characteristics of perforation and management*

Variable	Number (%)
Perforation at primary tumor site	
Yes	21 (24)
No	33 (37)
Unknown	35 (39)
Perforation site	
Colorectal	46 (52)
Small bowel	19 (21)
Stomach	3 (3)
Unknown	21 (24)
Median interval from last bevacizumab to perforation in weeks (range)	3.8 (< 1, 232)
Management	
Surgical	39 (44)
Non-surgical	50 (56)

^*^Numbers refer to number of patients and parentheses refer to percentages unless otherwise specified

**Fig. 1:**
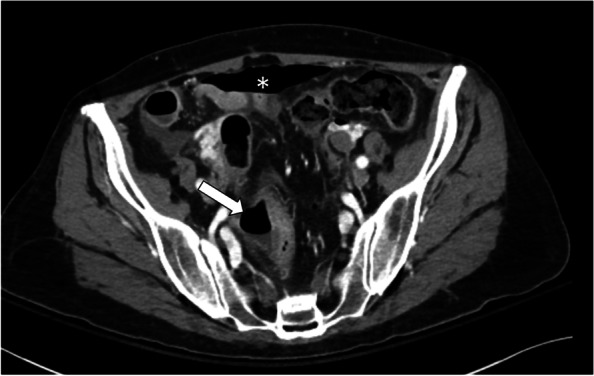
This axial contrast enhanced computerized tomography image of the pelvis shows an abnormal segment of rectosigmoid bowel with adjacent air (white arrow) and a moderate amount of free intra-peritoneal air (asterisk), all of which is consistent with a bowel perforation

Although gastric perforation is relatively rare, three patients had a well-documented perforation at this site. The first had been diagnosed with rectal cancer. Biopsies at surgery showed no tumor in the area of perforation but a perforated ulcer was seen and treated with an epiploic patch. This patient died from multi-organ failure in less than a week after the perforation. The second patient had metastatic ovarian cancer. At surgery, the area of perforation appeared friable and edematous with no gross evidence of tumor. The operative note described how the perforation seemed attributable to cancer therapy (bevacizumab), presumably because no other explanation was apparent. The perforation was approximated and sutured. The patient was not retreated with bevacizumab but went on to receive other systemic chemotherapy and died 5 months after the perforation. The third patient also had ovarian cancer. She was not surgically explored or treated but, on computerized tomography, had evidence of cancer in proximity to the perforation (Fig. 1). This patient was treated conservatively with nasogastric suction and pain medications and died within 2 months of the perforation.

### Non-surgical therapy

All patients were treated with supportive care and palliative therapy to treat symptoms and to address their other clinical needs. In 9 of these patients, a drain was placed by an interventional radiologist. In 4 patients, bevacizumab was resumed after the perforation, presumably after extensive patient counseling and presumably because of otherwise limited antineoplastic options.

### Survival after perforation

**Fig. 2:**
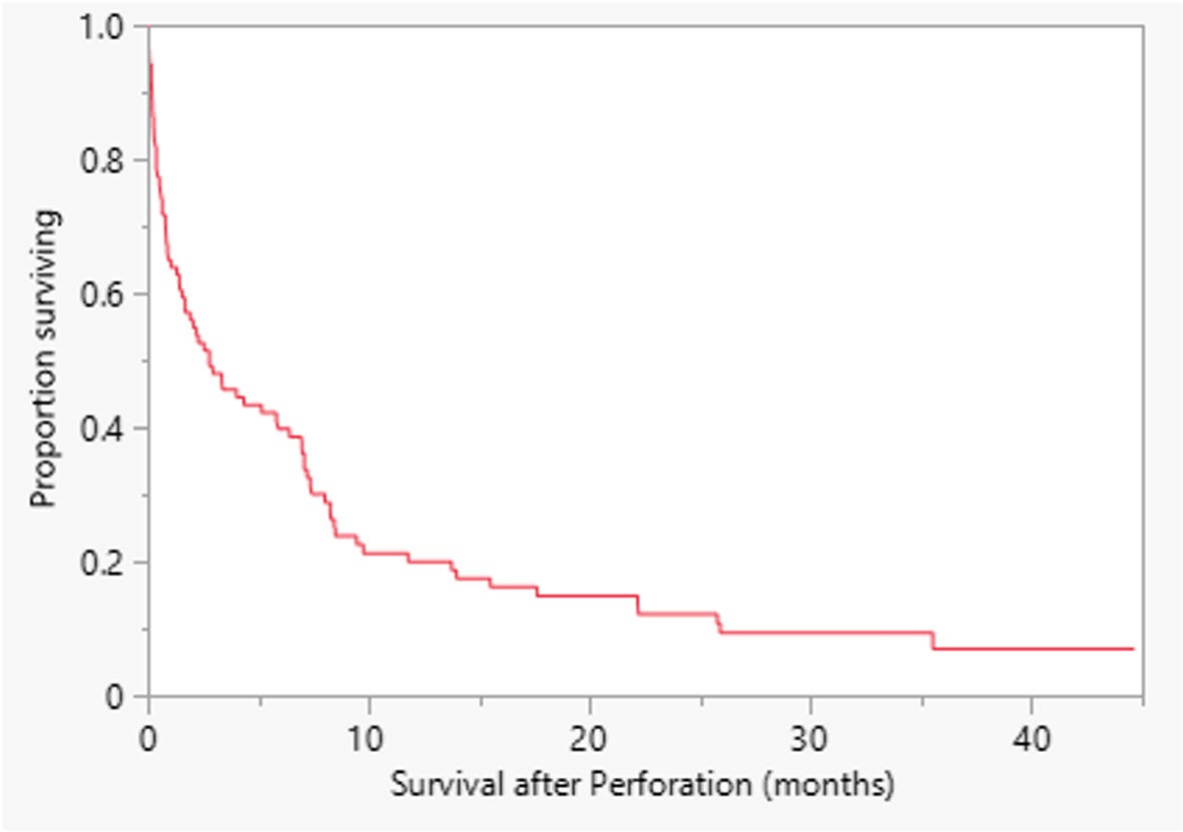
The median survival of all patients after perforation was 2.73 months (range 0–45 months) with 78 patients deceased at the time of reporting

Seventy-eight patients were deceased at the time of data analyses. The median survival of all patients after perforation was 2.73 months (range 0–45 months) (Fig. 2). Thirty-two of 89 patients (36%) died within 30 days of the perforation.

**Fig. 3:**
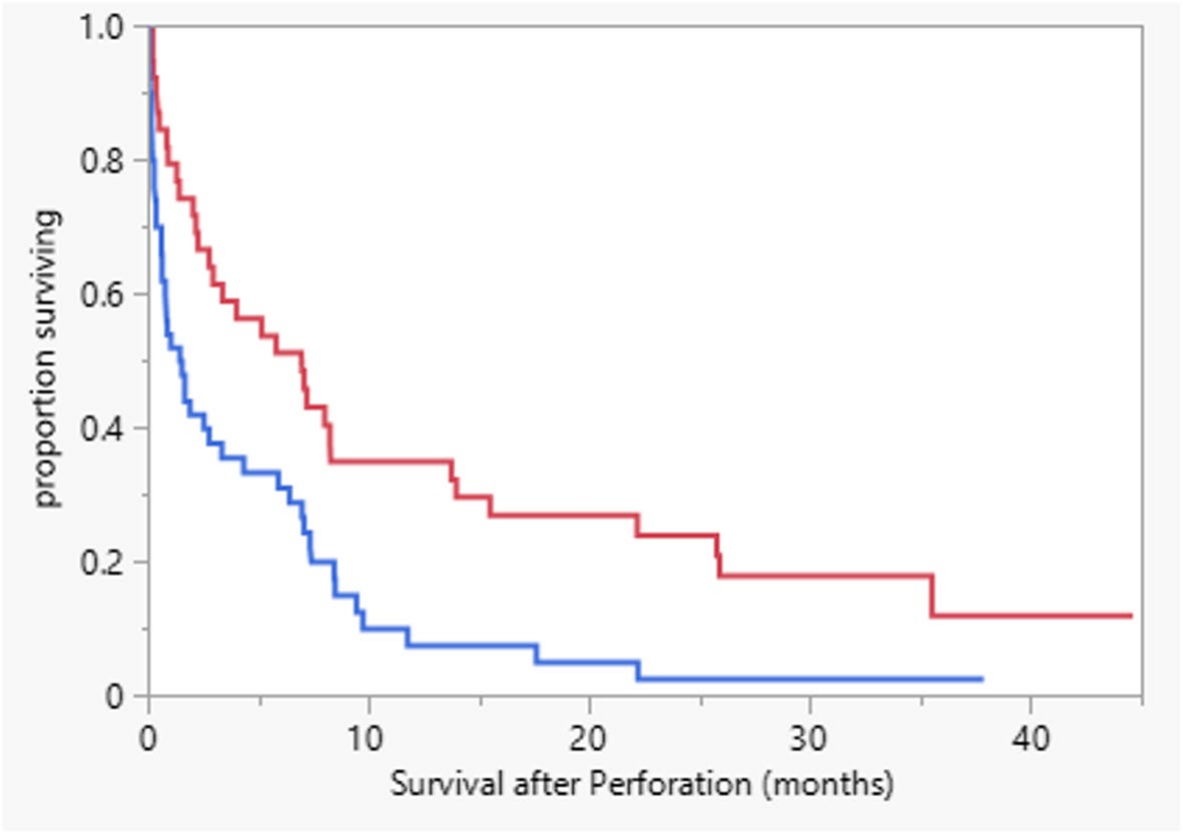
Surgical patients (red line) manifested a median survival of 6.9 months (95% confidence interval (CI) 2.2–13.6 months); whereas those who were not treated surgically manifested a survival of 1.45 months (95% CI 0.6–3.3 months), with a HR of 0.49 (95% CI 0.31–0.78); *p* = 0.003

Univariable models that examined age, gender, corticosteroid use, and time since last bevacizumab dose found no statistically significant associations with survival. In contrast, surgical patients manifested a median survival of 6.9 months (95% confidence interval (CI) 2.2–13.6 months); whereas those who did not undergo surgery manifested a survival of 1.45 months (95% CI 0.6–3.3 months), yielding a HR of 0.49 (95% CI 0.31–0.78) (Fig. 3 and Table 3). In multivariable analyses, surgical interventions were associated with improved survival, and corticosteroid use with worse survival (Table 3). Fewer than half of patients received systemic therapy after the perforation with the number of lines of therapy ranging from 0 to 5.

**Table 3:** Cox proportional hazards*

Variable	Univariable analyses		Multivariable analyses	
	Hazard ratio (95% confidence interval)	*p* value	Hazard ratio (95% confidence interval)	*p* value
Age at perforation (referent: incremental increasing age by year)	1.01 (0.99–1.03)	0.18	0.99 (0.99–1.03)	0.37
Gender (referent: male)	1.01 (0.62–1.62)	0.98	1.06 (0.64–1.76)	0.80
Corticosteroids (referent: no corticosteroids)	1.59 (0.94–2.67)	0.09	1.75 (1.02–2.99)	0.04
Time since last bevacizumab dose (referent: incremental time by weeks)	0.99 (0.99–.00)	0.82	1.00 (0.99–1.00)	0.37
Surgery (referent: no surgery)	0.49 (0.31–0.78)	0.003	0.47 (0.29–0.74)	0.002

^*^Smaller hazard ratios are indicative of longer survival

## Discussion

To our knowledge, this is the largest study to date to examine the overall median survival of patients who suffered from a gastrointestinal perforation after bevacizumab and to provide detailed patient-level data after the perforation. The current study provides important data that will be instructive for patients, their families, and healthcare providers on survival expectations with and without surgery. These data enable healthcare providers to inform patients and their families of survival outcomes and thereby allow for better decision-making. Other studies that had examined such overall survival outcomes included only small numbers of patients—24 and 7 patients, respectively, thus highlighting the importance of the current study [9, 10]. Other such studies examined perforation and fistula formation together and included only a small number of patients with a perforation (*n* < 10) [20]. Similarly, although the large database study from Wichelmann and others included 1375 patients with gastrointestinal perforation, these investigators reported only death rates, not overall survival and not whether surgery was undertaken [8].Thus, the overall survival data reported here provide a contemporary experience that enables patients, their families, and their healthcare providers to make better-informed management decisions and better informed expectations after the occurrence of this devastating drug-induced complication.

Gastric perforation from bevacizumab is relatively rare, possibly because of greater gastric wall thickness relative to bowel wall thickness. Because of the rarity of this adverse event, we provided more detail on the three patients who had a clear perforation in this anatomical location [8]. It is important to note that in one patient a non-malignant ulcer was present at the site of perforation. Such a finding suggests a role for ruling out peptic ulcer disease in patients at high risk or initiating ulcer treatment prior to starting bevacizumab. These findings also raise the possibility that it might be viewed as challenging to know for sure that the bevacizumab was truly implicated in these perforations.

The current study reports worse survival outcomes than previous studies. For example, Badgwell and others identified 24 patients with gastrointestinal perforation following treatment with bevacizumab and reported a 30-day mortality rate of 12.5%—in contrast to the 36% mortality rate reported here [17]. Another study reported that operative management of gastrointestinal perforation after bevacizumab yielded short-term mortality in 2 of 7 patients [18]. Three factors may explain the higher mortality reported in the current study. First, over time, the use of bevacizumab has become more restrictive; for example, it is no longer used in the adjuvant setting for colorectal cancer, whereas, in the distant past, some clinicians were prescribing this drug off-label for this indication [19]. It seems likely that patients who were able to receive postoperative (adjuvant) curative therapy with bevacizumab for colorectal cancer would be better able to withstand a major surgery after a perforation and or would be earlier in their disease course and have longer to live. In effect, these patients would have been cancer-free at the time of their second surgery, the recipients of far less chemotherapy, fitter, less likely to suffer surgical complications, and likely to have a longer lifespan. Because the current study provides a more contemporary experience, survival outcomes might be worse. Secondly, since these two earlier reports, new drugs, such as PDL-1 inhibitors and trifluridine and tipiracil, have received FDA-approval for the treatment of colorectal cancer and other malignancies [21, 22]. Again, more contemporary patients are receiving bevacizumab as a later-line systemic therapy. These conditions of treating patients further along in their cancer trajectory make it less likely that such patients would survive a perforation long-term because of advanced cancer and perhaps less likely that surgical options would even be considered. Third, 26% of patients in this study were receiving corticosteroids. It is not surprising that corticosteroids result in worse outcomes after a perforation, as these immunocompromised patients often have less dramatic symptoms at the time of perforation, have a delayed diagnosis of perforation, and therefore receive less punctual therapy. Fourth, the current study included over three times the sample size of the studies that preceded it and appears to be the largest to date to examine overall survival; thus, the higher mortality rate reported here might be more precise and meaningful.

Although this study observed that surgical interventions appeared to improve survival, this finding may, in part, reflect selection bias. Those patients more likely to achieve better outcomes and more likely to withstand a surgical procedure were perhaps more likely to be treated surgically. Although the current study showed better survival with surgery, a surgical approach should not be over-advocated under these circumstances. Currently, the decision of whether to pursue a surgical intervention after a gastrointestinal perforation after bevacizumab is determined on a case-by-case basis with no established management guidelines. Management of this drug-induced complication must consider the patient’s cancer diagnosis, cancer trajectory, and overall health with the survival data reported here providing an educational—rather than therapeutically defining—perspective on management options.

The current study has strengths and limitations. With respect to the limitations, first, we included patients who had suffered a perforation at any point following bevacizumab, with the majority having had their last dose within 7 weeks of the complication. Although some might consider this approach a limitation, by design, it enabled us to assess whether time from last dose of bevacizumab to diagnosis of perforation was predictive of survival (and we found it was not). Justification for this approach hinged on limited data that have characterized the architectural changes that led to the perforation and ongoing duration of such changes. Second, one might question the retrospective study design. The rarity of this complication points to the need to use such a study design. A prospective study design would be unlikely to generate the data presented here in a timely manner. Third, the fact that 26% of patients in the current study were receiving corticosteroids, which also predispose to perforation, is a confounding risk factor and should be duly noted. Further, it is possible that corticosteroids had an indirect effect by causing an ulcer, which in turn resulted in a perforation. Fourth, no study of this nature—or, for that matter of any other design—is able to state conclusively that a perforation is definitively attributable to bevacizumab. The sometimes long interval between bevacizumab administration and the perforation underscores this point. Nonetheless, these data provide information that could be of value for clinical care. Fifth, we do not know whether bevacizumab biosimilars were used. Future studies might focus on biosimilars. Finally, another limitation is that this study was unable to describe the exact site of perforation under all circumstances; again, this limitation as well as other clinical factors make it impossible to tease out cause and effect with regard to bevacizumab and perforation.

In terms of strengths, the more contemporary nature of this work is a major strength and increases the applicability of findings to current day patients. Similarly, as already noted, this appears to be the largest study to date to focus specifically on survival following a bowel perforation after bevacizumab administration. This relatively large, contemporary sample of patients enabled us not only to explore a set of relevant clinical variables as potential predictors of survival but also to report survival outcomes with greater confidence. Such survival outcomes will be of help in counseling patients and their families about whether surgical options should be considered or not.

In summary, this study characterized survival after a gastrointestinal perforation in patients who had received bevacizumab and provides important median survival data with surgery and without surgery. Although the outcomes reported here point to how a perforation is associated with diminished survival, it is important to point out that these are rare events and that, for example, in the experience reported here, over 2000 patients had been treated with bevacizumab. For the few times this devasting drug-induced complication occurs, the findings reported here can guide and explain outcomes to patients and their families as well as help healthcare providers, as management decisions need to be made.

## Data Availability

The data generated by this work is not available due to confidentiality.
